# Case Report: First report of peritoneal dialysis-related peritonitis caused by *Pseudomonas fulva*

**DOI:** 10.3389/fmed.2026.1844767

**Published:** 2026-05-25

**Authors:** Tian Feng, Yu Zhan, Yingmiao Zhang, Zhongxin Lu

**Affiliations:** 1 Department of Medical Laboratory, The Central Hospital of Wuhan, Tongji Medical College, Huazhong University of Science and Technology, Wuhan, China; 2 Hubei Provincial Engineering Research Center of Intestinal Microecological Diagnostics, Therapeutics, and Clinical Translation, Wuhan, China; 3 Cancer Research Institute of Wuhan, The Central Hospital of Wuhan, Tongji Medical College, Huazhong University of Science and Technology, Wuhan, China

**Keywords:** 16S rRNA sequence, case report, MALDI-TOF MS, peritoneal dialysis-related peritonitis, *Pseudomonas fulva*

## Abstract

*Pseudomonas fulva* (*P. fulva*) is a rare human pathogen, with few reported cases of infection caused by this bacterium. We report a case of an elderly patient with *P. fulva* infection who developed peritoneal dialysis-related peritonitis after peritoneal dialysis. A bacterial strain was isolated from peritoneal dialysis fluid and identified as *P. fulva* by matrix-assisted laser desorption ionization-time of flight mass spectrometry (MALDI-TOF MS) and 16S rRNA sequencing. The patient recovered after treatment with cefotaxime. A comprehensive review of *P. fulva* infections in pathogenic diagnosis, clinical treatment, and prognosis were presented in this paper.

## Introduction

*Pseudomonas fulva* (*P. fulva*) is a Gram-negative bacterium that was initially isolated from rice fields in Japan in 1963 (1). According to the study by Bartlett et al., this species is regarded as an established human pathogen (2). To date, there have been several cases of *P. fulva*-induced infection, including acute meningitis, ventriculitis, post-traumatic infection, cystitis, and septic shock (3–8). In some cases, *P. fulva* was primarily misidentified as *Pseudomonas putida,* which is a low-virulence opportunistic pathogen that causes nosocomial infections via medical devices (4, 5, 9). With the development of matrix-assisted laser desorption/ionization time-of-flight mass spectrometry (MALDI-TOF MS) and 16S rRNA sequencing, *P. fulva* can be accurately identified. In this report, we present the first case of peritoneal dialysis-related peritonitis caused by *P. fulva* and review the clinical characteristics of *P. fulva* infections based on previously reported cases.

## Case presentation

**Figure 1:**
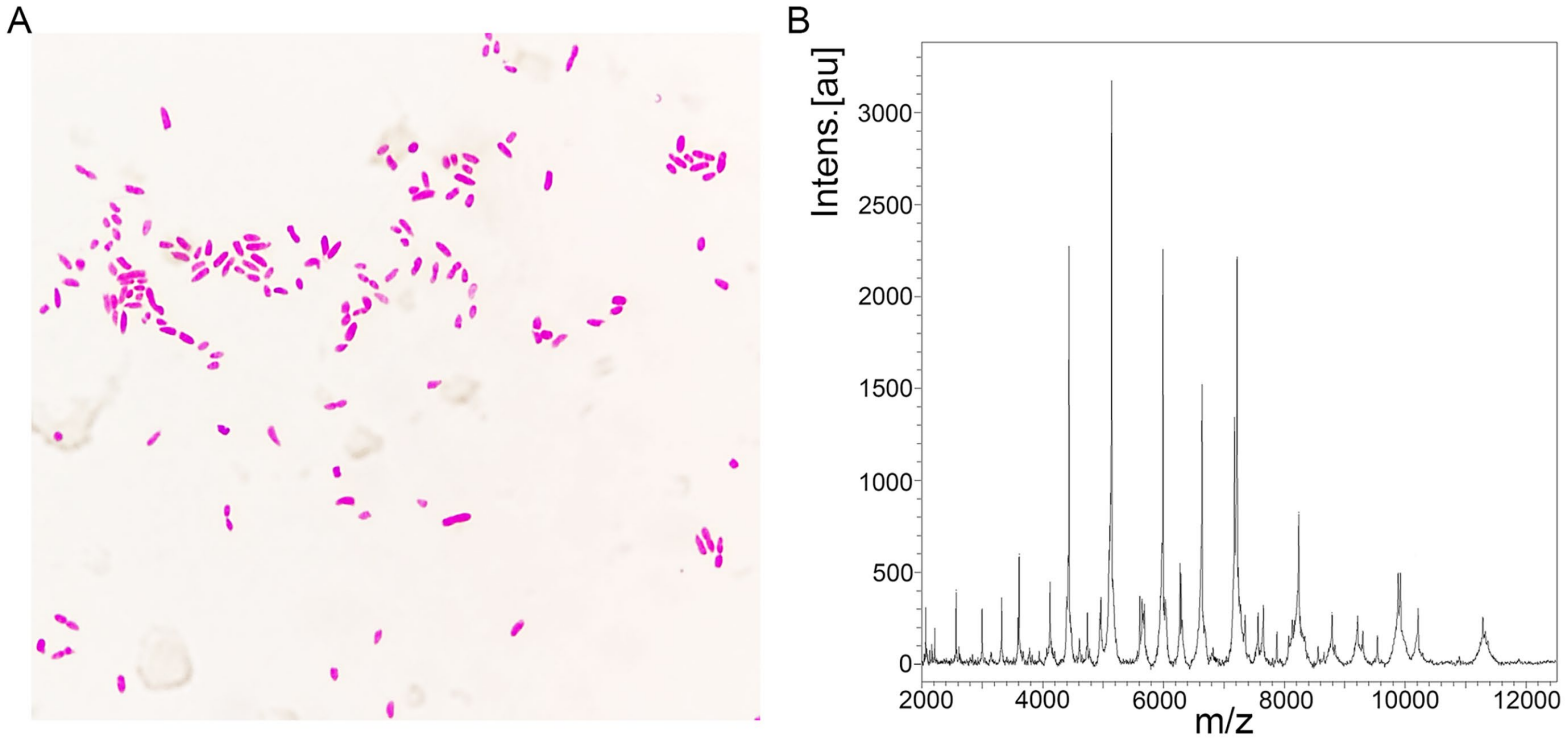
Isolation and identification of *Pseudomonas fulva* strain BL506. **(A)** Gram stain of the isolate. **(B)** The spectrogram of the isolate obtained by MALDI-TOF MS.

We present the case of a 67-year-old man with end-stage renal disease who was admitted to our hospital on 8 June 2022 and presented with progressive dyspnea as the chief complaint. The patient experienced persistent and worsening shortness of breath, which was the primary reason for seeking medical attention. He had a history of chronic kidney disease, diabetes mellitus, duodenal ulcer, congestive heart failure, pulmonary embolism, and hypertension. The patient had undergone peritoneal dialysis catheterization on 28 February 2022, as part of the previous management for end-stage renal disease. On 28 May 2022, during his ongoing home-based peritoneal dialysis treatment, he developed acute abdominal pain and discomfort while infusing peritoneal dialysis solution. The abdominal pain was relieved after temporary cessation of peritoneal dialysis. The patient, presenting with anorexia and postprandial vomiting, was admitted to the intensive care unit (ICU) of our hospital. Upon admission, the patient had a body temperature of 38.2 °C, a heart rate of 117 beats/min, pulse oxygen saturation (SpO_2_) of 91–98% (nasal cannula oxygen), blood pressure of 156/100 mmHg, and a respiratory rate of 30 beats/min. The serological and biochemical parameters of the patient were also presented (Supplementary Table S1). On June 10, the patient’s body temperature increased to 38.3 °C. Relevant laboratory tests showed a white blood cell (WBC) count of 15.61 × 10^9^/L (87.1% neutrophils) and a procalcitonin level of 0.21 ng/mL. The laboratory received samples for routine ascites analysis and two sets of ascites cultures. To minimize contamination and ensure the accuracy of pathogen detection, ascitic fluid specimens were collected using standardized aseptic techniques. Upon collection, samples were immediately transferred into sterile, disposable vacuum blood collection tubes without anticoagulants, labeled with patient identifiers, and transported to the clinical laboratory for pathogen testing within 1 h. If immediate processing was not feasible, specimens were stored at 4 °C for a maximum of 24 h to prevent bacterial overgrowth or mortality, thereby avoiding potential discrepancies in test results. The ascites was dark brown and turbid, with a WBC count of 2.60 × 10^9^/L [95% polymorphonuclear leukocytes (PMNs)]. After 18 h of aerobic culture, both vials tested positive. The Gram-negative bacilli were visible under the microscope (Figure 1A). Subsequently, the positive cultures were inoculated onto Columbia blood agar, MacConkey agar, Chocolate agar, and Sabouraud agar (Guangzhou Dijing Microbial Technology Co., Ltd., Guangzhou, China) incubated at 35 °C in the presence of 5% CO_2_. After overnight incubation, smooth, moist yellow colonies were observed on Columbia blood agar. The isolate BL506 was identified as *P. fulva* using the MALDI-TOF MS Biotyping System (Bruker Daltonik GmbH, Germany) following a series of pretreatment steps according to the manufacturer’s instructions (Figure 1B).

**Figure 2:**
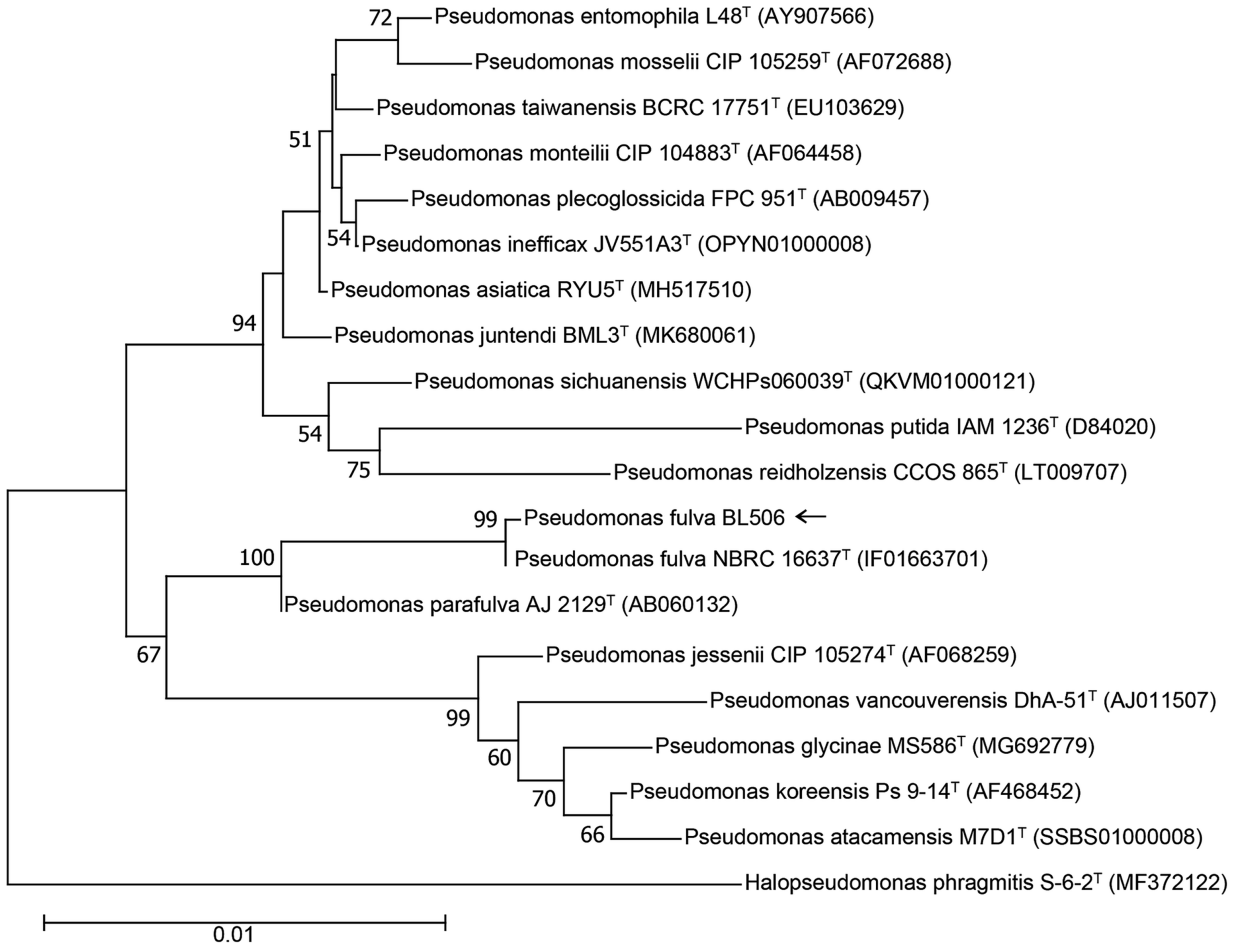
Phylogenetic tree based on the 16S rRNA gene sequences showing the relationship between isolated strain BL506 (black arrow) and other members within the genus *Pseudomonas*. The tree was reconstructed using the neighbor-joining method, with *Halopseudomonas phragmitis* S-6-2^T^ (MF372122) as an outgroup. Bootstrap values (>50%) from 1,000 replicates are indicated at branch nodes. ^T^, type strain.

The 16S rRNA gene sequence of the strain BL506 was analyzed using common 16S rRNA primers (forward primer: 5’-ATTGAACGCTGGCGGAG-3′; reverse primer: 5’-GTGATTCATGACTGGGGT-3′). A total of 1,425 nucleotides were obtained. The 16S rRNA sequence was analyzed with the 16S-based ID on EZbiocloud (https://www.ezbiocloud.net/identify). The sequence analysis showed 99.37% identity with the 16S rRNA gene of the type strain *P. fulva* NBRC 16637^T^ (GenBank accession number: IF01663701), and this cluster was strongly supported by a bootstrap value of 99% (Figure 2). Combined results from MALDI-TOF MS and 16S rRNA sequencing confirmed the isolate as *P. fulva*, and the patient was diagnosed with peritoneal dialysis-related peritonitis.

The antimicrobial susceptibility testing (AST) was conducted using the AutoMic-i600 automatic microbial identification and drug susceptibility analyzer (Autobio Diagnostics Co., Ltd., China) (Table 1). The strain was sensitive to all antibiotics tested. Based on the AST results, the patient was treated with intravenous cefotaxime. After 4 days of antibiotic therapy at 1 g every 12 h, the patient’s complete blood count (CBC) and body temperature returned to normal. The patient was subsequently discharged after 10 days of admission.

**Table 1:** Antimicrobial susceptibility of *Pseudomonas fulva* BL506.

Antibiotics	MICs (mg/L)	Category^a^
Amikacin	≤ 4	S
Aztreonam	8	S
Cefepime	1	S
Ceftazidime	≤ 1	S
Ceftriaxone	≤ 8	S
Ciprofloxacin	≤ 0.5	S
Gentamicin	≤ 2	S
Imipenem	≤ 0.5	S
Levofloxacin	≤ 1	S
Meropenem	1	S
Piperacillin/tazobactam	≤2/4	S
Cefoperazone/sulbactam	≤16/8	S
Tobramycin	≤ 1	S

^a^S, susceptible.

*Pseudomonas fulva*-related PD peritonitis is a rare infection caused by environmental opportunistic pathogens. The primary risk factors in this case are ESRD in the uremic phase (severe renal insufficiency), hypokalemia, PD catheter-related barrier defects, environmental/operational contamination, and flora disturbance induced by broad-spectrum antibiotics. The patient’s body temperature and complete blood count returned to normal after anti-infective therapy, indicating that the infection was primarily caused by exogenous environmental bacterial invasion and that the antibiotics were sensitive, leading to a good prognosis.

## Discussion

*P. fulva* is a Gram-negative environmental bacterium originally isolated from rice fields. A majority of prior studies on this bacterium are in the field of agriculture, focusing on its bioremediation capabilities (10, 11). For example, Adeniji et al. proposed that *P. fulva* could serve as a candidate biocontrol agent for maize fusariosis—a plant disease caused by *Fusarium* species (12). It has been more than a decade since *P. fulva* was first reported to cause human infections, and there are nine reported cases of *P. fulva*-associated infections (10, 11) (Table 2).

**Table 2:** Case reports in *Pseudomonas fulva*-related infections.

No.	Year	Country	Sex	Age	Symptoms	Sample	Identification	Diagnosis	Treatments	Outcome	Reference
1	2010	Argentina	F	2	Fever, malaise	CSF	16S rRNA	Ventriculitis; neuroectodermal tumor	AMX, SXT, VAN, MEM, COL, CRO, TZP, tuberculostatic drugs	Died	(3)
2	2010	Korea	M	56	Fever	blood	16S rRNA, MLSA	Sepsis	TZP, CLI	Survived	(4)
3	2014	USA	F	55	Fever	CSF	16S rRNA	Ventriculitis	LEV, Rifampin	Survived	(5)
4–19	2014	China	*F* (4), M (12)	33–91	N/A	N/A		Sepsis	AMC, AZI, CXM, CMZ, CSL, FOX, LEV, MOX, OFX, TZP	Survived (15) Died (1)	(9)
20	2016	Spain	M	73	Swelling, erythema	wound exudate	MALDI-TOF MS	Skin and soft-tissue infection	TEC, FEP, CAZ	Survived	(6)
21	2018	Pakistan	M	45	Fever	blood	MALDI-TOF MS	bacteremia	TZP, VAN	Died	(19)
22	2021	Sweden	F	85	Hematuria, dysuria	urine	MALDI-TOF MS	Cystitis	PME	Survived	(7)
23	2023	Japan	M	93	Fever, chills, and shivering	blood	MALDI-TOF MS	Septic shock	TZP, PIP	Survived	(8)
24	Present case	China	M	67	Fever, abdominal pain		MALDI-TOF MS, 16S rRNA sequence	Peritonitis	CTX	Survived	

M, male; F, female; CSF, cerebrospinal fluid; MLSA, multi-locus sequence analysis; MALDI-TOF MS, matrix-assisted laser desorption ionization-time of flight mass spectrometry; AMX, amoxicillin; SXT, trimethoprim/sulfamethoxazole; VAN, vancomycin; MEM, meropenem; COL, colistin; CRO, ceftriaxone; TZP, piperacillin/tazobactam; CLI, clindamycin; AMC, amoxicillin/clavulanic acid; AZI, azithromycin; CXM, cefuroxime; CMZ, cefmetazole; CSL, cefoperazone/sulbactam; FOX, cefoxitin; LEV, levofloxacin; MOX, moxalactam; OFX, ofloxacin; TEC, teicoplanin; FEP, cefepime; CAZ, ceftazidime; PME, pivmecillinam; PIP, piperacillin; CTX, cefotaxime; N/A, not accessible.

These cases confirm that *P. fulva* is a human pathogen, and cases of *P. fulva* infection are increasing. With the advancement of detection technologies such as MALDI-TOF MS and 16S rRNA sequencing, an increasing number of *P. fulva* infection cases are expected to be reported. Rapid diagnostic tools, including MALDI-TOF MS, which has been demonstrated to accelerate microbiological identification in peritoneal dialysis-related peritonitis, are similarly valuable for guiding early targeted antimicrobial therapy in *P. fulva* infections (13). Furthermore, the antibiotic resistance profile of *P. fulva* is a challenge for clinical treatment, highlighting the urgent need for further investigations into the pathogenicity and epidemiological characteristics of *P. fulva* infections.

Rapid and accurate pathogen identification is crucial for optimizing clinical decision-making in infectious diseases and oncology. Although traditional culture-based methods remain the gold standard, they have inherent limitations, such as prolonged detection times and the inability to detect fastidious or unculturable microorganisms. Over the past few decades, three advanced technologies, including MALDI-TOF MS, 16S rRNA sequencing, and next-generation sequencing (NGS), have addressed these shortcomings and revolutionized clinical diagnostics.

MALDI-TOF MS is currently widely used in clinical practice; however, this technique remains highly dependent on traditional microbial culture and the isolation of pure bacterial strains (14). In contrast, 16S rRNA gene sequencing exhibits greater advantages in diagnosing culture-negative bacterial infections. A study by Rampini et al. demonstrated that their molecular 16S broad-range PCR assay showed >90% concordance with routine bacterial culture (the gold standard) when tested on 394 clinical specimens (15). For an additional 231 clinical specimens of various types from patients with culture-negative bacterial infections, this assay achieved a sensitivity of 42.9%, a specificity of 100%, a positive predictive value (PPV) of 100%, and a negative predictive value (NPV) of 80.2% (15).

Notably, the high conservation of the 16S rRNA gene severely limits its ability to discriminate between closely related species—a limitation shared with MALDI-TOF MS (16). In polymicrobial infections, 16S rRNA gene sequencing further fails to accurately quantify the abundance of individual species or distinguish pathogenic bacteria from commensal flora (16). Low-biomass samples are particularly susceptible to reagent contamination; studies have shown a relatively high detection rate of contaminants (e.g., *Propionibacterium acnes*) in negative control samples, and contamination in high-biomass samples may even mask the signals of true pathogens (16). Another limitation of 16S rRNA gene sequencing is its inability to differentiate between viable and dead bacteria (17), as it only detects DNA presence. This can result in the overestimation of pathogen burden; for example, residual DNA from non-viable bacteria following antibiotic treatment can still be detected, complicating the assessment of clinical treatment efficacy. Furthermore, 16S rRNA gene sequencing data cannot directly reflect microbial metabolic activity or virulence gene expression (16), necessitating complementary functional verification via metagenomic or metabolomic approaches.

In comparison, NGS enables whole-genome analysis of multiple pathogens in a single run, whether applied to clinically isolated bacterial strains or metagenomic samples (18). A key advantage of NGS over 16S rRNA gene sequencing is that a single experimental protocol can be universally applied to the identification and typing of all pathogens (16, 18). Therefore, in clinical practice, pathogen identification requires a context-specific approach; clinicians should evaluate the specific scenario and select the most appropriate diagnostic method based on practical clinical requirements.

## Data Availability

The original contributions presented in the study are included in the article/Supplementary material, further inquiries can be directed to the corresponding author/s.
