# Supplementary material for: Case Report: First report of peritoneal dialysis-related peritonitis caused by Pseudomonas fulva
Source: Front Med (Lausanne). 2026 May 25;13:1844767. doi: 10.3389/fmed.2026.1844767 (PMC13243026; doi:10.3389/fmed.2026.1844767)
Supplement: Supplementary file 1 [file Table_1.DOCX]

Supplementary Table 1. Serological and biochemical parameters of the patient.

| **Parameter** | **Result** | **Unit** |
| --- | --- | --- |
| Creatinine (Cr) | 361.9 | μmol/L |
| Urea | 12.64 | mmol/L |
| Sodium (Na^+^) | 141.3 | mmol/L |
| Potassium (K^+^) | 3.13 | mmol/L |
| Chloride (Cl^-^) | 97 | mmol/L |
| Hepatitis B surface antibody | 31.04 | mIU/mL |
| Anti-HCV antibody | 0.17 | S/CO |
| Anti-HIV antibody | 0.13 | S/CO |
